# Editorial: Gender dysphoria: Diagnostic issues, clinical aspects, and health promotion

**DOI:** 10.3389/fpsyt.2022.1000939

**Published:** 2022-10-12

**Authors:** Maria Inês Rodrigues Lobato

**Affiliations:** ^1^Serviço de Psiquiatria, Hospital de Clínicas de Porto Alegre—HCPA, Universidade Federal do Rio Grande do Sul (UFRGS), Porto Alegre, Brazil; ^2^Programa de Pós-Graduação (PPG) Psiquiatria e Ciências do Comportamento, Universidade Federal do Rio Grande do Sul (UFRGS), Porto Alegre, Brazil; ^3^Coordenadora Programa de Identidade de Gênero—PROTIG, Universidade Federal do Rio Grande do Sul (UFRGS), Porto Alegre, Brazil

**Keywords:** gender dysphoria, transsexual, mental health care, medical approach, gender identity

During the years 2021 and 2022, I had the opportunity to edit a supplement of the journal Frontiers of Psychiatry / Section Public Mental Health on the theme Gender Dysphoria—American Psychiatry Association / Gender Incongruence—World Health Organization, which was a very rewarding experience.

Since 1998, we have been working on the development of assistance and research protocols with the purpose of finding the best medical care for this group of people. Our work has always aimed toward being an agent of change in our city, state, and country. Once a socially stigmatized theme, is now discussed as a medical, social, and cultural phenomenon, relevant both in the academic community and to the population in general, largely due to the unprecedented nature and seriousness of our work since the beginning (1–3).

One example of this is that, through our patients and the Federal Public Ministry, we have provoked the regulation of medical assistance by the Unified Health System (SUS) throughout Brazil.

Over the years, we have become a reference center in Latin America for professional training and research and have graduated several doctors and masters in the medical, psychological, social work, and speech therapy fields. Our program (*Programa de Identidade de Gênero—PROTIG*) has evaluated more than one thousand patients and we have performed more than 300 gender-affirming surgeries.

Although we have come a long way, we still have many questions to be answered in order to improve the quality of life of these people in terms of diagnostic procedures, therapeutic indications, and prognostic factors.

Different results of research focused on clinical aspects of people in care with a diagnosis of Gender Dysphoria were published in this supplement.

The study presented by Silva et al. sought to relate physical and sociodemographic characteristics to quality of life in individuals undergoing hormonal treatment, and it found no difference in relation to quality of life (QoL) between transsexual men and women and found that altered gender affirmative physical aspects favor the quality of life of all patients.

Garcia et al. focused on understanding aspects of the family functioning of our patients, and found a prominent absence of the father figure in family relationships in both transsexual men and women and identified the need for care services to prioritize educational programs for families.

Silva et al. presented the results of their research about sociodemographic and psychosocial aspects in transsexual people associated with negative outcomes such as ruminative thinking. They found higher scores of ruminative thinking in transsexual women, and this outcome was related to high levels of stress, anxiety, depression, and suicidal ideation.

Soll et al. studied the population of young people between 8 and 16 years old who were under follow-up at PROTIG (2014) and found that 45% of young transsexuals were positive for psychiatric comorbidity during their lifetime.

Moisés da Silva et al., through the retrospective research of 214 medical records, sought to understand more clearly the surgical aspects of patients submitted to sexual affirmative surgical procedures, namely postsurgical complications, and concluded that the vaginoplasty procedure using the penile inversion technique is relatively safe, promoting functionality and personal satisfaction.

Villas-Bôas et al. sought to identify the main process of voice in transsexual women and concluded their voices were feminized through glottic adaptations.

Guadagnin et al. using an online protocol to monitor patients during the pandemic period, found a greater deterioration in their social vulnerability when compared to the pre-pandemic period (4).

A follow-up study conducted in Toronto-Canada by Singh et al., with boys clinically referred for gender identity concerns in childhood, reported a high rate of “desistance” for gender dysphoria and a high rate of a biphilic/androphilic sexual orientation. Singh et al. also stressed that the implications of the data for current models of care for the treatment of gender dysphoria in children should be discussed.

We conclude that our supplement may provide knowledge-generating ideas and suggestions for a better medical approach to people with gender dysphoria.

## Author contributions

The author confirms being the sole contributor of this work and has approved it for publication.

## Funding

This work was funded by Fundo de Incentivo à Pesquisa e Eventos do Hospital de Clínicas de Porto Alegre (FIPE/HCPA), Fundação de Amparo à Pesquisa do Estado do Rio Grande do Sul (grant number INCT/FAPERGS: 17/2551-0000519-8), National Council for Scientific and Technological Development (CNPq), Coordination for the Improvement of Higher Education (CAPES), and Postgraduate Program in Behavioral Sciences, Psychiatry at UFRGS.

## Conflict of interest

The author declares that the research was conducted in the absence of any commercial or financial relationships that could be construed as a potential conflict of interest.

## Publisher's note

All claims expressed in this article are solely those of the authors and do not necessarily represent those of their affiliated organizations, or those of the publisher, the editors and the reviewers. Any product that may be evaluated in this article, or claim that may be made by its manufacturer, is not guaranteed or endorsed by the publisher.

## References

[1] Bränström R Pachankis JE. Reduction in mental health treatment utilization among transgender individuals after gender-affirming surgeries: a total population study. Am J Psychiatr. (2020) 177:727–34. 10.1176/appi.ajp.2019.19010080 31581798

[2] Dhejne C Vlerken RV Heylens G Arcelus J. Mental health and gender dysphoria: a review of the literature. Int Rev Psychiatr. (2016) 28:44–57. 10.3109/09540261.2015.1115753 26835611

[3] Fontanari AMV Rovaris DL Costa AB Pasley A Cupertino RB Soll BMB. Childhood maltreatment linked with a deterioration of psychosocial outcomes in adult life for southern Brazilian transgender women. J Immigr Minor Health. (2018) 20:33-43. 10.1007/s10903-016-0528-6 27838863

[4] Guadagnin F da Silva DC Schwarz K Villas Bôas AP Lobato MIR. The impact of the COVID-19 pandemic on the lives of people with gender dysphoria. Front Public Health. (2022) 10:878348. 10.3389/fpubh.2022.878348 35874999 PMC9298877

